# The Buffalo Medical College

**Published:** 1883-08

**Authors:** 


### THE BUFFALO MEDICAL COLLEGE.

We have received the thirty-eighth annual announcement of this institution, which now ranks among the oldest of our medical colleges, and also one of the best in the ability and high professional character of the men who, in the past, have honored it with their labor and influence. It will be seen, by referring to the advertisement on the fourth page of our cover, that several important changes have taken place in its faculty. Within a few years the institution has suffered the loss by death of Prof. White, the ablest didactic instructor in obstetrics in this country. On account of physical disability, Prof. Miner has been compelled to relinquish the chair of special and clinical surgery. His name now honors the institution by standing at the head of its faculty as emeritus professor. Another severe blow, quite unexpected and involving a loss which it will be impossible to supply, is the resignation of Prof. E. M. Moore, who for many years has been a tower of strength to the college, attracting from far and near, by his eminent ability and wide reputation as a surgeon and teacher, students who have loved him for his sterling worth and unsullied private and professional character. The retirement of three such men within a brief period, makes a void which it will be difficult, if not impossible, to fill, and the college must feel the effect which such losses inflict.

Prof. Moore's resignation is a loss to the profession of this city as well as to the college. It is but fair to say of him that he is recognized as the ablest lecturer on surgery in this country. In the clearness of his lectures and the logical and practical arrangement of the subject-matter he presents, and his large and varied experience, Prof. Moore has but few equals and no superior. We join, therefore, in the universal regret felt among the friends of the college and in the profession, that circumstences have arisen which have led him to sever his relations with the college, and to discontinue a life-habit of teaching in which he has achieved such eminent success and popularity.

We learn that Dr. Roswell Park, of Chicago, has been appointed lecturer on surgery and clinical surgery in the place thus vacated. We fail to ascertain, after repeated inquiries in surgical circles, that the new appointee brings to this responsible position any very extensive experience or reputation. Dr. Park enters upon his new duties a comparative stranger, whom we do not know, and who, if he possesses the ability to fill the chair once occupied by men of such surgical eminence as Hamilton and Moore, should possess a high order of professional attainments, an extensive experience in his profession and such skill as an operator as to make himself known and become a valuable acquisition to the profession of this city. The position is a difficult one to occupy and will test the metal of the new incumbent.

Among the new lecturers, we notice with pleasure the names of Dr. D. W. Harrington, as clinical lecturer on surgery at the Sisters of Charity Hospital, and Dr. W. H. Heath of the Marine Hospital Corps, as lecturer on genito-urinary diseases. These gentlemen are active and earnest workers in their special departments and will prove valuable instructors.

The faculty now presents many new names, mostly strange to our readers and but little known to the profession at large.

We regret that the college fails to place itself *en rapport* with the current of the best medical thought of the country and at once inaugurate a graded course of study, an examination for admission and other important features so generally called for by the profession in the interests of higher education. It possesses the opportunity now to take an advanced position. Quality rather than quantity, in its matriculants and graduates, would enhance its reputation, and make it subserve a noble purpose for the advancement of the great interests of the medical profession, rather than for the furtherance of private professional and pecuniary interests.

